# Adverse effects of political sanctions on the health care system in Iran

**DOI:** 10.7189/jogh.05-020302

**Published:** 2015-12

**Authors:** Roxanne L Massoumi, Sumana Koduri

**Affiliations:** Medical College of Wisconsin, Milwaukee, WI, USA

The United States has long leveraged economic sanctions as powerful instruments to achieve foreign policy objectives [1]. Economic sanctions have been described as a “cheaper form of coercion, less aggressive than war with fewer human costs, and more politically feasible” [1]. Sanctions may be implemented as tariffs on imported goods, quotas on how much can be imported or exported, embargoes that prevent all trade between countries, or non–tariff Barriers that include other non–tariff restrictions on imported items [2]. No matter what type of sanction is used, the end goal is the same: to force a change in behavior [2]. The United States particularly favors investment withdrawals, trade embargoes and foreign aid reductions to coerce foreign countries into compliance with its foreign policy objectives. However, many question the effectiveness of economic sanctions and, while policy–makers debate this, what is often understated is the hidden cost of US diplomacy: the cost to the citizens of the country in question, particularly on their health care sector. The goal of this viewpoint is to bring attention to the impact that economic sanctions can have on the health care system in the impacted nation.

## IRAN

One such nation that has been crippled by economic sanctions is Iran. The political sanctions, set on Iran since 1979, have attempted to spare the average citizen from harm by specifically excluding the health care system from trade restrictions. However, the health care system is indirectly affected by the sanctions, as we have learned from personal interviews with physicians in Iran and by reading locally published articles during the summer of 2013.

One effect of the economic sanctions is that the purchase of health care supplies must be done via a currency transfer that has become difficult and unreliable between Iran and the western world [3]. The *Economist* writes that “many companies and financial institutions remain reluctant to trade with Iran for fear of penalties,” and that this is likely to be affecting the importing of goods, including medicine [3]. The Iranian professor and pharmacist, Cheraghali, writes [4]: “Although almost all sanctions in the recent decades had provisions for exemptions of medicines and food stuffs, sanctions (through complications in transportation, difficulty in transferring hard currencies or either lack of capital) commonly lead to disruption of health services and even basic nutrition of the ordinary people in the sanctioned countries.”

Having difficulty trading with the West, the Iranian government is currently trading with its eastern allies. This exchange is indeed helpful for Iran to obtain basic supplies, however, many newer medications are still under a pharmaceutical patent and not being manufactured outside of the United States or Europe, limiting Iran’s access to them [5]. Some supplies are being manufactured within Iran, which is a limited method of manufacturing, as the limit on currency transfer has made importing starting material such as active pharmaceutical ingredients and finished products difficult and often impossible for Iran [4]. The sociologist S. A. Hosseini writes [6] that the “quality of pharmaceuticals and treatment of patients have been affected due to changing the sources of imported medicines and raw materials for locally produced pharmaceuticals.”

A pediatric surgeon from one of Iran’s largest public academic hospitals expands on this when he expresses his understanding, based on personal experience, that “the sanctions were not supposed to be imposed on medicine, however, because it has become difficult to transfer money to pay for drugs, the sanctions have indirectly affected medicine. Approximately 50–60% (a personal estimate) of drugs cannot be manufactured in Iran because there is also a lack of starting materials. Suturing material, for example, is in shortage and is now either made in Iran or bought from China, both of which yield lower quality sutures (in the physicians experience).” An ENT surgeon from a private day surgery clinic adds that Iran is “coping by getting the drugs at a more expensive price from other countries, such as Dubai. Some drugs, [they] cannot even get from anywhere.” These are the personal opinions of the contacts interviewed in Iran and not based on official reports.

Faced with a decreased access to medication [4,6,7], Iran’s health care professionals are burdened with the unenviable task of determining where, and to whom, their resources should be allocated. While resources are typically allocated based on need, with fatal diseases and emergency surgery as the highest priority, supplies are limited even for patients with the greatest need. An ENT surgeon describes his thoughts on Iran’s current situation: “Things are harder and some drugs are no longer found. Transplant and cancer patients suffer the most because of the lack of drugs. Things that are scarce are instruments, like positron emission tomography machines, and drugs, like chemotherapy and anesthesia.”

Conversations with two Iranian anesthesiologists paint an even more lucid picture of the adverse effects of the medicine shortage in Iran. An adult anesthesiologist in a private practice cited remifentanyl and isoflurane as two of the more common drugs for which there was a shortage of supply. At a public academic hospital, a pediatric anesthesiologist commented on the medicine shortage and provided an example of how the shortage directly affects the Iranian children: “Sevoflurane is used to induce when a child is agitated, but it is expensive so we often use halothane instead. Halothane is an older drug and does not induce anesthesia as quick, however, it is cheaper and more available now.” Aside from being outdated, halothane may lead to severe side effects, such as hepatic necrosis (which is most often unpredictable and not dose–dependent). It should be used as a drug of last resort when newer medication is unavailable.

**Figure:**
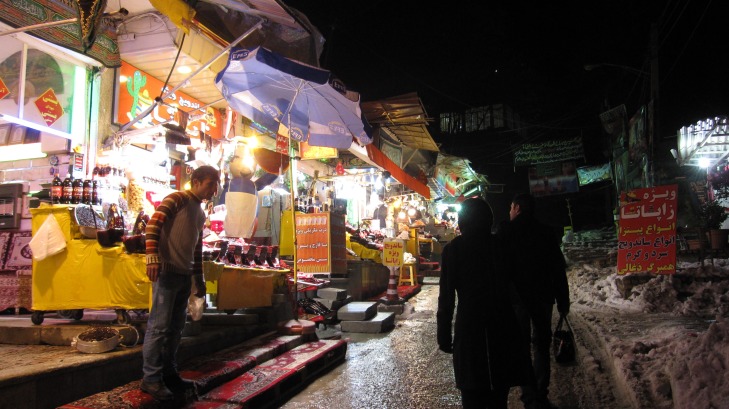
Photo: Courtesy of Roxanne L Massoumi, personal collection

In addition to a shortage of resources, Iran has also been isolated from the international research community. Recently, the Dutch publishers Elvesier sent a note to their network of US based editors and reviewers encouraging them to “avoid handling manuscripts if they include an author employed by the government of Iran” [8]. The pediatric surgeon mentioned before addresses his personal experience with this: “… academic research has been affected, as it has become harder to publish articles from Iran; the articles are less likely to be accepted into academic journals.” Iranian researcher, Saeidnia, gives further insight into Iran’s isolation from the global academic community when explaining that there are “many problems with money transaction in order to do scientific activities, for example [for] payment of publication fees, society subscription fees, or registration fees in international congresses” [9]. This is consistent with Cheragali’s claim presented above that the health care system is being indirectly affected because of the limitations on currency transfer [4].

## IRAQ

When regarding Iran’s current situation, it begs the question: is Iran an isolated occurrence, or have sanctions adversely affected other countries’ health care sectors with regularity and been ignored? The neighboring country of Iraq’s health care system was affected by the US–led economic sanctions implemented on them from 1990–2003 [10]. Much like the situation in Iran, the economic sanctions in Iraq, which were believed to have spared its health care system, adversely affected it through the inability to transfer currency and a resulting shortage of medicine and medical instrumentation. According to the scientist Clare Sansom, “Doctors and technical staff found it almost impossible to keep even these [cobalt 60 radiotherapy] machines in service during the sanctions, because of import restrictions, difficulties with equipment manufacturers, and bureaucracy,” and she describes a lengthy waiting list for treatment of tumors, even those as severe as intracranial neoplasms [11].

Iraq did not recover quickly once the sanctions had been lifted and, over a decade later, their health care sector remains unstable. Today it is considered “weak, with non–functioning equipment, inadequate drug supplies, and a fragile infrastructure” [10]. While this is a complex issue involving many factors, the instability that sanctions created in the health care system are a major contributing factor. Sansom [11] notes Iraq’s position in the global medical sphere: “Iraq has been almost completely isolated from the international community for more than a decade; medical journals and even textbooks were unavailable, and there were no opportunities for doctors to travel abroad.” Medicine continued its globalization throughout the 1990s, but Iraq, with its weak infrastructure, was excluded from sharing in the international community’s knowledge. As a result, Iraq’s health care sector continues to lag behind as those of other countries rapidly advance.

## POST–SANCTION REBULDING AND THE UTILITY OF SANCTIONS

Both Iran and Iraq had health care systems that deteriorated as an indirect result of political sanctions. It is important to note the difficulty Iraq’s health care system had in recovering from sanctions, as we foresee Iran traversing the same, rough road. Although the Iranian and Iraqi governments are markedly different, Iran will need to take similar steps as Iraq to rebuild their infrastructure. Iraq was ill equipped and unable to take these necessary steps, and by bringing Iraq’s struggle to light, a similar fate may be avoidable for Iran.

A post–sanctioned era in Iran may be in the near future and policy–makers should begin devising a plan now that would help physicians provide better care as soon as restrictions on resources are lifted for Iran. The negative costs of sanctions on the average citizens of a country are very real and must be strongly considered prior to implementing the sanction. While they may be effective in pushing governmental policies, sanctions negatively impact common citizens and their health care, as seen in both Iran and Iraq. Cheraghali argues [4]: “There is now consensus among political scientists that the record of sanctions in achieving their stated objectives is low. Instead, ordinary people who live in sanctioned countries have to bear costs attributed to the sanction.” While foreign policy objectives often leave no alternative to economic sanctions, politicians and their constituents, alike, should be cognizant of the negative effect sanctions can have on health care systems.
